# Can a ‘consent to contact’ community help research teams overcome barriers to recruitment? The development and impact of the ‘Research for the Future’ community

**DOI:** 10.1186/s12874-019-0843-4

**Published:** 2019-10-22

**Authors:** Katherine Grady, Martin Gibson, Peter Bower

**Affiliations:** 1 Research & Innovation, Salford Royal NHS Foundation Trust, Salford, UK; 2 NIHR Clinical Research Network, Greater Manchester, UK; 3 NIHR Clinical Research Network and Centre for Primary Care and Health Services Research, University of Manchester, Greater Manchester, UK

**Keywords:** Clinical trials, Electronic health records, Data linkage, Recruitment database

## Abstract

**Background:**

Recruitment to health research remains a major challenge. Innovation is required to meet policy commitments to help patients take part in health research. One innovation that may help meet those policy goals is the development of ‘consent to contact’ systems, where people give generic consent to be contacted about research opportunities. Despite their potential, there are few empirical assessments of different ways of recruiting patients to such communities, or of the value of such communities to local research teams.

**Main text:**

We describe the development of the ‘Research for the Future‘consent to contact community, outline the recruitment of patients to the community, and present data on their participation in research.

**Discussion:**

Over 5000 people have been registered across 3 clinical areas. A range of recruitment strategies have been used, including direct recruitment by clinicians, postal invitations from primary care, and social media. In a 1 year period (2016–2017), the community provided over 1500 participants for a variety of research projects. Feedback from research teams has generally been positive.

**Summary:**

The ‘Research for the Future‘consent to contact community has proven feasible and useful for local research teams. Further evaluation is needed to assess the cost-effectiveness of different recruitment strategies, explore patient and researcher experience of its advantages and disadvantages, and explore how the community can be more reflective of the wider population.

## Background

The NHS White paper set out a proposal to ensure that patients are made aware of research studies relevant to them [1], and the NHS Constitution outlined guiding principles for the promotion, conduct and use of research to improve population health [2]. The NHS Operating Framework (2012/13) confirmed research is a core NHS function [3], and the Government’s plan for growth encouraged NHS providers to improve clinical research performance by implementing measures around ‘recruitment to time and target’ [4]. Despite such proposals, research teams often struggle to recruit the numbers required in the time available [5–8].

A survey of the public’s attitude towards health research showed high levels of confidence in NHS research and willingness to participate [9]. However many people are unaware of what research takes place locally or how to get involved. For example, many people with common disorders such as diabetes are managed in primary care whilst research centres are often based in specialist settings.

The challenge of recruitment has encouraged innovation in patient recruitment. One innovation that could have major implications for recruitment to health research is the use of ‘consent to contact’ models.

### Consent to contact

Using information in medical records to find suitable patients may offer a solution to recruitment difficulties – a view supported by NHS England mandate 2016/17 which recommended better use of technology to support research, innovation and growth [10].

However, under the Data Protection Act 1998, using health records for research should ideally obtain prior informed consent from the individual, especially following the introduction of the General Data Protection Regulation (GDPR). One way of doing this is to develop registers of patients who have given prior consent, who can be approached by researchers with appropriate studies. This is described as ‘consent to contact’, and is defined by the Academy of Medical Sciences as a ‘mechanism … for individuals to give generic consent to be contacted about suitable research opportunities, before considering whether they consent to take part in a specific study’ [11].

Consent to contact models raise a number of ethical, regulatory and organisational issues, and a number of examples have been published with different features [12–16]. The question ‘does a central registry for members of the public (i.e. a list of people with contact details) who are interested in taking part in a randomised trials improve recruitment?’ was recently identified as one of the top 20 research questions relating to how best to improve the process of recruiting people to randomised trials [17]. In this paper, we describe a model developed in Greater Manchester, provide data on its operation and impact on recruitment, and outline its advantages and disadvantages. The aim is to make a contribution to the developing evidence base around consent to contact models and their role in health research.

## Main text

‘Research for the Future’ is a collaboration between NIHR Clinical Research Network (CRN): Greater Manchester, North West EHealth, Northern Care Alliance NHS Group and Health Innovation Manchester. It consists of a series of ‘Help BEAT’ campaigns, of which diabetes was the first. Each ‘Help BEAT’ campaign invites people with a particular health condition to register their details and research interests as part of a research community. In doing so, they give their ‘consent to contact’ and can be approached in the future about a range of research opportunities. The main ethical issues for the community concern ensuring that information is secure, up-to-date, and that patients can choose to leave the community if they find it burdensome.

In this paper, we aimed to describe the ‘Research for the Future’ consent to contact community, how patients are recruited into the community, and the effectiveness of different methods of recruitment. We also describe the characteristics and research interests of people in the community; and the engagement of people within the community in research.

Our aims were as follows:

- To describe the ‘Research for the Future’ community and its development. We provide a short narrative description of the development of the community over time, and some of the core functionality in the system.
- To describe recruitment into the community, and the impact of different methods. We present data on the growth of the community over time, and preliminary data on the effectiveness and costs of different methods of attracting new volunteers to register.
- To describe the characteristics of people registered with the community. We present demographic and clinical characteristics of the volunteers.
- To describe the engagement of people in the community in research. We describe patterns of engagement of enrolled individuals, outline the different types of studies they have participated in, and offer reflections on the experience of using the system to support research studies.

The discussion summarises the current status of the Research for the Future community, outlines its advantages and disadvantages, and considers future developments.

### The development of the ‘Research for the Future’ website and data systems

Research for the Future involves both a website (https://www.researchforthefuture.org/) which provides patients with a way to register securely, as well as a bespoke data system.

The project was set up late 2011 as a partnership between Greater Manchester Comprehensive Local Research Network, the Diabetes Research Network and North West E-Health. Current partners in the project and the staff involved can be found on the website (https://www.researchforthefuture.org/).

At that time, only about 40% of commercial and non-commercial studies on the local NIHR portfolio were recruiting to time and target. The pilot project was set up to test new strategies to enable people who want to be involved in research to quickly access studies. Diabetes was chosen as the pilot because local studies were struggling to recruit. Implementation of diabetes guidelines resulted in people previously managed in secondary care (where the research centres were located) being discharged to primary care.

The project aimed to deliver improvements in recruitment to diabetes studies through setting up a database and creating a publicity campaign, ‘Help BEAT Diabetes’, to increase awareness about diabetes research.

The objectives of the pilot were:

- To set up a database so that people who register their interest via the Help BEAT Diabetes campaign can be contacted directly about specific studies they were eligible to take part in
- Develop a communications strategy to maintain volunteer engagement and interest in the campaign through the development of a website and promotional materials
- Demonstrate that the contact centre and database can be used to recruit volunteers to diabetes portfolio studies.

The Research for the Future website was revised and updated when the pilot expanded to include respiratory and heart disease campaigns. Content was based on the original website but additionally incorporated feedback from the community. As part of the evaluation of the pilot in 2014, volunteers were invited to participate in a feedback survey. This included questions on frequency of website use, feedback on content (e.g. most/least useful pages) and suggestions for improvement. Over 300 responses were received. Findings revealed those who had visited the website found it easy to navigate with interesting content. However, almost two thirds of respondents had never visited the website. With the introduction of additional clinical areas, the project team used the pilot evaluation feedback to inform website development, such as simplifying the website address and revising content.

Disease specific questions on the website registration form are based on common research protocol criteria, approved by Clinical Research Network staff and clinical leads. Partner organisation North West EHealth ensured that the design and delivery of the ‘eligibility checker’ page and online registration form conformed to appropriate information governance standards to protect patient confidentiality. The site was designed as a flexible platform where additional Help BEAT campaigns could be added in the future. The website went live June 2016 and the version currently in use won an award for design in the NIHR Let’s Get Digital Awards 2017 competition.

Maintenance of the patient register is required to ensure it remains current. Staff follow-up all emails and any post that is returned. To confirm that database details remain current, their NHS number is used to check their demographic details against the NHS spine data every 3 months (the spine is NHS Digital’s infrastructure for health and social care in England which joins together information across local and national NHS systems).

We have chosen to refer to Research for the Future as a ‘community’, as this is a more user friendly term than ‘register’ or ‘database’, which may have particular connotations. Although the ‘community’ functions of Research for the Future are limited, volunteers have the opportunity to interact with the database team, and potentially each other via our Facebook page. They are also invited to relevant health/research events throughout the year, and all receive a newsletter twice a year. Website development also saw creation of an area for researchers and volunteers to share their stories to encourage others to get involved (‘case studies’ section of each microsite, https://www.researchforthefuture.org/cs-research-area/diabetes/).

### Recruitment of people into the community, and the effectiveness of different methods

Currently 5587 people are registered on the Research for the Future community, and 3981 (71%) live in the target area (corresponding to the NIHR CRN: Greater Manchester footprint).

**Fig. 1:**
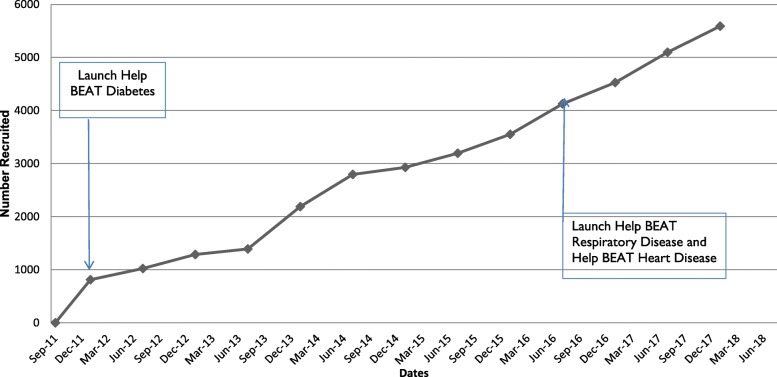
Recruitment into the community

Figure 1 shows the recruitment numbers since initiation of the project and the launch of subsequent ‘Help BEAT’ campaigns.

### Methods of recruitment onto the community

A variety of methods are used to recruit patients to the community. NHS staff can recruit patients during routine visits. A number of general practices have recruited using mailed invitations to their patients, which involves use of FARSITE software (https://nweh.co.uk/uploads/documents/FARSITE-Frequently-Asked-Question.pdf) to identify and mail patients with an invite to participate. Patients have also been recruited through charity partners, or have registered directly. A recent focus has been the use of social media (especially Facebook) to encourage registration directly with the website.

NHS staff are the source of most registrations (29%), while an additional 21% are recruited through general practice using FARSITE. Self-registration through the campaign website accounts for 11% of registrations, and charity partners account for another 11%. The remaining registrations come from radio advertising (8%), social media (7%), poster/leaflet (4%) and other routes such as newspaper/magazine articles; other websites, seeing the promotional video and word of mouth (9%).

The team have undertaken analysis of the costs of some of the recruitment methods, compared to their yield in terms of recruits to the community. In terms of GP recruitment, 18 practices have invited 5981 patients to join Help BEAT Diabetes, of whom 339 have signed up (5.6%) at a cost of £3466 (£10.22 per registration). For radio advertisement, we placed two 12 week adverts (one specifically targeted diabetes, one for a range of conditions). Cost for both radio advertisements was £24,750, and they generated 482 responses (£51.35 per registration). In terms of social media, we initially placed 37 adverts or boosted posts, generating 208 registrations at cost of £14 per registration. Further roll-out and implementation of the social media strategy has reduced this to £9.50 and £4.50 respectively in later social media cycles. Since the project commenced, 593 have left or been removed from the community (average 8 per month).

### Types of people in the community

Table 1 shows demographic details of people registered. The initial version of the database did not record ethnicity, which meant that it was difficult to explore the representativeness of the registered patients compared to the regional population. Recent enhancements have now enabled this to be recorded.

**Table 1:** Demographic details of people registered with the community

Category	All patients	Diabetes	Respiratory	Heart disease
Gender				
Male	2990 (53.5%)			
Female	2594 (46.4%)			
Other	3 (0.05%)			
Age				
Mean age	59.4	59.1	61.3	66.9
Range	18–98	18–96	22–89	19–96
Condition^a^				
Diabetes	4960	Type 1 28.0% (*n* = 1389)	COPD 53.2% (*n* = 324)	Myocardial infarction 37.8% (*n* = 177)
Respiratory	609	Type 2 64.0% (*n* = 3174)	Asthma 49.9% (*n* = 304)	Angina 27.5% (*n* = 129)
Heart	468	NDH 7.9% (*n* = 391)	Bronchiectasis 8.5% (*n* = 52)	Atrial Fibrillation 22.9% (*n* = 107)
		Other 0.1% (*n* = 6)		Heart Failure 12.0% (*n* = 56)

^a^some people are registered on more than one community

### Engagement in research

Table 2 shows the types of studies members of the community say they are interested in (from a list that people are given at registration). There was no clear preference for observational research or studies involving interventions.

**Table 2:** Research Interests of individuals in the community

Research type	*n* =	%
Clinical trials –medical equipment	5523	98.9
Observational studies	5307	95.0
Questionnaires - postal	5286	94.6
Interviews – face to face	5210	93.3
Interviews – telephone	5041	90.2
Clinical trials –existing treatments	5013	89.7
Clinical trials – new treatments	4981	89.2
Focus Groups	4836	86.6
Questionnaires - electronic	4400	78.8

In a recent 12 month period (2016–2017), the community helped to recruit participants to 22 clinical studies (599 patients) and 11 non-clinical studies (1000 patients). Studies included questionnaires, focus groups, observational studies and clinical trials. It is not possible at this point to determine what proportion of patients in the community participated in studies, as some patients may participate in multiple studies, and their activity is not tracked.

We seek feedback from research teams who engage with Research for the Future, in order to understand how the community has helped their research, and what types of invitations are more and less successful. Generally, our experience is that better responses are obtained to studies with the least strict inclusion criteria, complexity and duration of commitment required from the participant.

Although many of the invitations are to take part in research, Research for the Future is also used to find participants to work alongside research teams in patient and public involvement. We have occasionally found that novel research models attract significant interest. For example, patients reported that studies that provided them with relevant test results were attractive in providing a level of engagement compared with studies where they were more passive recipients. Researchers report that Research for the Future can also be very useful to gather large number of responses for questionnaire studies.

## Conclusions

### Summary of main findings

Our consent to contact community has registered over 5000 patients in three disease areas, and has facilitated the recruitment of over 1500 patients to a variety of research studies. We suggest that the data presented here demonstrates that the community is feasible and potentially useful. A clearer assessment of its value will require additional data and comparisons with alternatives.

### Strengths and weaknesses of data

There are some limitations to the data presented here. Data collection has been a priority for the Research for the Future project, to support ongoing development and evaluation, but resources are limited as there is no funding for independent evaluation. We have feedback from researchers about the service in the form of quotes, but we have not reported those here as they are potentially selective. We have found it difficult to obtain information from research teams on the number of volunteers the community has provided once their recruitment is complete. Funding is required to enable us to better understand how the consent for contact community is used and the experiences of patients and researchers using the community.

Data reported on the ‘cost per registration’ of different methods are based on the most complete cost data we have available, but are not formal economic costings, and should be treated as illustrative only.

Costings for the different recruitment strategies provide some indication of their relative yield and have informed ongoing discussions about recruitment, but we have been unable to assess their value in terms of eventual research participation – for example, it is possible that a particular method is responsible for greater numbers of registrations, but that may not translate into greater levels of subsequent research participation. If there was sufficient interest in the potential of systems such as Research for the Future, formal research could provide more accurate data on these issues, as well as exploring the experience of patients and professionals through detailed qualitative research.

Targeted marketing from a respected health care professional or organisation is the most successful method of attracting people to the database, but is clearly dependent on engagement from clinical professionals, which may be variable and difficult to sustain in time-pressured clinical environments [18, 19]. Recruitment through general practices can provide fairly consistent yield, but overall numbers are low. Social media has proven to be efficient, although this may raise additional concerns about the representativeness of patients recruited through such methods. Our experience has highlighted the importance of a recognised and trusted brand, and the role of the NHS branding in helping people decide they want to register.

Some important data of interest to the development of consent to contact communities (such as the proportions of patients who engage with research in any time period) cannot be gathered through our systems at present.

### Strengths and weaknesses of the ‘consent to contact’ model

All methods used to recruit participants to health research (including clinician referral) have potential selection bias, and it will be important to assess the relative bias that might exist within this community compared with other methods. Obviously, one way of reducing bias is to increase the numbers of patients registered in the community, together with ongoing assessment of the degree to which the registered patients differ from local and national populations on the conditions of interest.

### Next steps and future research question

A consent to contact community model is feasible and useful but little is known about the mechanisms by which patients decide to register with such communities. Qualitative research could usefully explore these issues to better inform later recruitment strategies, alongside issues of cost. There is also the interesting issue of why patients leave, and how engagement can be maintained. At present, engagement with community members is achieved through the website (which includes an area to share stories and feedback from research teams), social media and a twice yearly newsletter. Retention of patients in individual studies is an under-researched area [20], and we know less about what would retain patients in a research community.

Engaging with patients in the community may be crucial to ensuring ongoing registration, both in terms of providing plenty of opportunities for research, but also providing other benefits such as information on health and research more generally, as well as opportunities to engage with the wider community. It will also be important to explore the optimal level of engagement with patients over time, and assess the impact of both a lack of opportunities (which may lead to disillusionment), and excessive opportunities (such may lead to patient burden and fatigue). At present, our experience would suggest that it is the former which is more of an issue.

An interesting issue is the ‘protectiveness’ of some local research leads regarding their patients who regularly participate in their research, which can lead to reluctance to promote the community to them, in case it limits their participation in studies run by those local teams. To overcome these concerns, we have developed the system with functionality to restrict invitations to people to any new study whilst they are already participating in an existing one.

Earlier we highlighted the issue of the representativeness of patients in the community. Clearly, increasing the proportion of patients registering with Research for the Future will help to allay concerns. An important question is whether it is possible to make the offer of registration on Research for the Future part of the routine process of taking part in any local research. If this was considered feasible and acceptable, it would also be important to explore the optimal time for doing so - for example, should it be part of the consent process, or would it be better as an offer at the end of participation in a research project? We are exploring the feasibility of this model locally.

### Summary

Recruitment to health research remains a major challenge. There is a need for innovation to meet policy commitments to increase opportunities for patients to participate in high quality research. We have presented data on the development of Research for the Future, and outlined the developments that are needed to maximise its contribution to meeting those aims.

## Data Availability

The data that support the findings of this study are not publicly available, as permission to share the data more widely has not been sought from patients.

## Abbreviations

GDPR: General Data Protection Regulation
NIHR CRN: National Institute of Health Research Clinical Research Network
